# Inpatients’ opinions on a hospital in Portugal

**DOI:** 10.12688/f1000research.2-49.v1

**Published:** 2013-02-13

**Authors:** Carina C Silva, Agripino Oliveira

**Affiliations:** 1 Department of Internal Medicine, Centro Hospitalar Vila Nova Gaia, Espinho, Portugal

## Abstract

**Background:** Little is known about the relationship between the opinions of inpatients and the degree to which hospitals are improving in performance over time. The aim of this study was to determine the personal assessment level of inpatients or their representatives regarding aspects of health care in an internal medicine ward.

**Methods:** We carried out a questionnaire in September 2011 with 284 discharged patients and patient representatives, focusing on their opinions about the department, health professionals and amenities, with response options ranging from 1 (very bad) to 5 (very good). The relationships between domains from the questionnaire and socio-demographic factors were examined using a t-test and one-way ANOVA.

**Results:** The response rate was 78%. The patients showed a slightly higher mean score (m) for factors in the medical care domain than did the patient representatives (m = 4.51 vs. m = 4.27; p = 0.014). The mean score of all the items in all domains was 4.24; this allowed us to determine the difference from the overall mean (DIFM) for medical care (DIFM = 0.18; p = 0.000), foods (DIFM = –0.31; p = 0.000), diagnostic tests (DIFM = –0.15; p = 0.036) and transport (DIFM = –0.41; p = 0.000). Respondents with a medium or higher educational level gave lower scores to the domains food (m = 3.74; p = 0.004), diagnostic tests (m = 3.72; p = 0.04) and transport (m = 3.62; p = 0.025) than those with lower educational levels. The domains facilities (m = 2.4; p = 0.04) and diagnostic tests (m = 3.63; p = 0.009) were given lower scores by those aged <50 years compared with older respondents.

**Conclusions:** Our findings suggest that the evaluation of the responders will allow the hospital management to make improvements in the quality of care.

## Introduction

Patient satisfaction has been increasingly used as a quality indicator in health care ^1^. The theoretical concept of satisfaction is controversial, but the user relates it with the set of reactions experienced ^2^. Therefore, the measurement of customer satisfaction should be considered as a personal opinion of health care services that are provided. One of the most used measures ^3^ is the difference between the expectations of the user in relation to the care and their perception of the care actually received. Indeed, it is expected that the patients, throughout their experiences, build a set of beliefs about the health system and professionals. The importance of attending to this type of belief has implications for the quality of communication with health professionals, the degree of trust in health care service delivery and customer satisfaction with the care provided. The aim of this study was to determine the personal assessment of inpatients or their representatives regarding aspects of health care in an internal medicine ward.

### Setting

The Centro Hospitalar Vila Nova Gaia-Espinho, where the study was conducted, is divided into several units. Patients admitted into the internal medicine department who need diagnostic imaging or invasive procedures are transported by ambulance to the central unit.

## Materials and methods

Several sources and methods were used to determine the questions to be included in the questionnaire. Firstly, a search was conducted using the Medline database with the aim of evaluating the tools that have been developed so far to assess the satisfaction of patients hospitalized ^4^. Secondly, focus groups of patients, caregivers and health care professionals were used to explore opinions about the positive and negative aspects of care received during hospitalizations. These focus groups were geared towards understanding the issues and expressions that could be used to develop questions to be included in the questionnaire. Thirdly, we developed a pool of items, based on the results of the focus groups and literature search, to be included in the questionnaire. These items were tested with a group of patients and health professionals, and they gave their opinions about the appropriateness of the items and the skills needed to comprehend them and evaluate the content and face validity of the questions.

The questionnaire was designed with twenty-two closed questions. It captures ten domains selected by their relevance to the study: the department’s image, facilities, medical care, nursing care, health care assistants (HCAs), secretarial services, reception, food, diagnostic tests and transport. Each domain is composed of between one and four discrete items rated on a five-point scale, in which the response options range from 1 (very bad) to 5 (very good) as shown in Appendix 1. The score for each domain represents the mean of the responses to each item within a given domain. The global mean score was determined by the sum of the items divided by the number of items answered. The questionnaire also contained socio-demographic variables, such as gender, age, educational level, occupational status, marital status ^4^ and type of respondent.

The respondents’ understanding of the questionnaire items can generate different opinions for each one of them. This diversity exposes the problem of internal consistency of the questionnaire, that is, the degree of uniformity between the answers to each item of the questionnaire. This internal consistency can be measured by Cronbach’s alpha ^5^, which varies from 0 to 1 ^6^, and the higher the count, the greater the reliability of the scale of questionnaire. A value of at least 0.70 reflects an acceptable reliability, between 0.80 to 0.90 moderate to high, and exceeding 0.90 high internal reliability.

### Study participants

From 1 ^st^ to 30 ^th^ September 2011, the self-administered questionnaire was filled out by the discharged patients or their representatives. All patients admitted more than 48 hours were given the questionnaire and an envelope. Family members or caregivers (referred to as ‘representatives’) replaced patients with severe physical or mental diseases, who would have difficulty in understanding and filling out the questionnaire. The deceased were excluded because their representatives would be in mourning.

All participants were informed of the study’s objective, and it was explained to them how to fill out the questionnaire. Delivery was carried out in a sealed envelope. To ensure confidentiality, participants were asked to put the completed questionnaires in a closed box at the time of discharge, according to the declaration of Helsinki. The board of directors and ethics committee of the hospital approved the study.

### Statistical analysis

We describe the frequencies (number), percentages, means (µ), median and standard deviation (σ) of the variables. In univariate analysis, we applied the Student’s t-test and analysis of variance (ANOVA) for the domains addressed in the questionnaire, considering the value of p < 0.05 statistically significant. Statistical analysis was performed using Statistical Package for the Social Sciences (SPSS) version 19.

## Results

A total of 284 inpatients were enrolled for the study, of whom 199 completed the questionnaire (response rate = 78%) and 31 died (10.9%). Respondents had a mean age of 62.9 years with a median of 66.5; 51% were men; 64% were married or cohabitating; 59.3% were retired and 51.7% had a basic (primary) education ( Table 1).

**Table 1.** Sociodemographic data of the respondents.

Variables		Number	%	Mean	Median
**Age [years]**				62.87	66
**Length of stay [days]**				12.26	9
**Sex**	Women	98	49		
	Men	101	51		
**Marital status**	Married/Cohabitating	126	64		
	Single	23	11.7		
	Widowed	31	15.7		
	Separated/divorced	17	8.6		
**Occupation**	Employed	45	22.7		
	Unemployed	15	7.5		
	Homemaker	12	6		
	Student	2	1		
	Retired	118	59.3		
	Other	7	3.5		
**Education level**	No education	10	5.9		
	Primary studies	89	51.7		
	High school/secondary education	58	33.7		
	University	15	8.7		
**Length of hospital** **stay**	< 9 days	101	50.8		
	9 to 13 days	40	20.1		
	> 13 days	58	29.1		
**Respondents**	Patient	133	66.8		
	Family	61	30.7		
	Caregivers	3	1.5		
	Other	2	1		
**Age Group**	< 50 years	44	22.2		
	50–65 years	53	26.8		
	> 65 years	101	51		

Cronbach’s alpha measures of internal consistency were computed for each of the ten domains, which showed a reliability for the overall scale of 0.89 ( Table 2). In all domains, acceptable values were met, reaching a minimum of 0.868 (for diagnostic tests) and a maximum of 0.880 (for medical care).

**Table 2.** Grouping of domains with their respective Cronbach’s alphas.

Domain	Cronbach’s alpha
Department’s image	0.881
Facilities	0.874
Medical care	0.888
Nursing care	0.879
Nursing assistants	0.875
Secretarial services	0.880
Reception	0.885
Food	0.881
Diagnostic tests	0.868
Transport	0.882
Cronbach’s alpha GLOBAL	0.890

**Figure 1.**
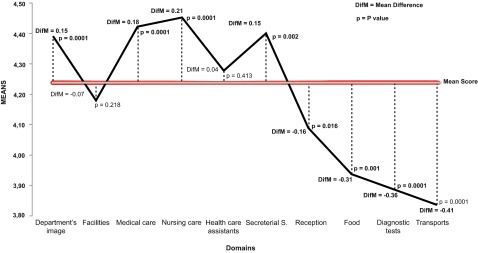
Ratio of the mean global score to the mean from the different domains of the questionnaire. Student t test or analysis of variance (ANOVA) with the Tukey’s multiple comparison test performed.

Answers to the items in the ten domains had an overall mean score of 4.24 ( Figure 1), out of a maximum of 5. This allowed us to compare with the mean score for each domain. The following domains had significantly more positive scores than the overall mean: department’s image (mean difference (DIFM) = 0.15; p = 0.0001), medical care (DIFM = 0.18; p = 0.0001), nurses (DIFM = 0.21; p = 0.0001) and secretarial services (DIFM = 0.15; p = 0.002). The following domains had significantly more negative scores than the overall mean: reception (DIFM = –0.16; p = 0.016), food (DIFM = –0.31; p = 0.0001), diagnostic tests (DIFM = –0.15; p = 0.036) and transport (DIFM = –0.41; p = 0.0001). In the domains related to the facilities and HCAs, there were no significant differences from with the overall mean.

Table 3 shows a univariate analysis of selected variables and their relationship with the mean scores of the questionnaire’s domains. We found that the variables ‘gender’, ‘occupation’, ‘marital status’ and ‘length of hospital stay’ showed no difference in all domains, except for secretarial services, for which higher scores were given by married respondents (µ = 4.49; p = 0.008).

**Table 3.** Univariate analysis by relevant variable.

		Depart. image		Facilities		Medical care		Nurs. care		HCAs		Secretarial S.		Reception		Food		Diag. testes		Transports	
		µ	σ	µ	σ	µ	σ	µ	σ	µ	σ	µ	σ	µ	σ	µ	σ	µ	σ	µ	σ
Gender	Men	4.41	0.59	4.16	0.8	4.38	0.66	4.45	0.66	4.32	0.6	4.4	0.6	4.11	0.84	3.99	0.75	3.88	0.9	3.88	1.06
	Women	4.37	0.58	4.18	0.69	4.46	0.63	4.46	0.66	4.24	0.6	4.39	0.74	4.06	0.87	3.87	0.84	3.89	0.8	3.78	0.95
	*P value*	0.681		0.852		0.387		0.984		0.367		0.862		0.69		0.29		0.947		0.51	
Respondent	Patient	4.49	0.52	4.2	0.72	4.51	0.59	4.49	0.64	4.34	0.58	4.39	0.7	4.11	0.84	3.91	0.82	3.92	0.81	3.83	1.02
	Family	4.19	0.65	4.13	0.8	4.27	0.73	4.37	0.7	4.17	0.62	4.42	0.61	4.03	0.9	3.99	0.76	3.83	0.91	3.85	0.98
	*P value*	***0.001***		0.525		***0.014***		0.222		0.059		0.735		0.57		0.496		0.483		0.932	
Education level	No/primary	4.4	0.6	4.28	0.71	4.4	0.68	4.51	0.68	4.39	0.58	4.43	0.62	4.29	0.73	4.1	0.82	4.	0.79	4.	0.97
	Sec./university	4.39	0.56	4.08	0.78	4.49	0.59	4.38	0.59	4.17	0.57	4.35	0.72	3.89	0.83	3.74	0.76	3.72	0.92	3.62	1.05
	*P value*	0.915		0.075		0.332		0.181		***0.013***		0.467		***0.002***		***0.004***		***0.04***		***0.025***	
Marital status	Marrid./cohabita.	4.43	0.53	4.21	0.74	4.47	0.63	4.44	0.67	4.29	0.61	4.49	0.61	4.07	0.91	3.93	0.82	3.87	0.87	3.84	1.04
	Si/div./widowed	4.31	0.66	4.09	0.76	4.34	0.68	4.45	0.65	4.26	0.57	4.21	0.75	4.08	0.76	3.93	0.77	3.88	0.78	3.8	0.94
	*P value*	0.15		0.278		0.17		0.949		0.755		***0.008***		0.946		0.958		0.94		0.807	
Work status	Employed	4.23	0.66	3.95	0.82	4.38	0.73	4.33	0.67	4.17	0.60	4.34	0.68	3.85	0.82	3.95	0.70	3.83	0.93	3.74	1.14
	Retired	4.42	0.55	4.23	0.72	4.42	0.64	4.48	0.67	4.29	0.58	4.45	0.68	4.14	0.89	3.98	0.82	3.95	0.78	3.90	0.95
	Other	4.49	0.56	4.30	0.70	4.53	0.54	4.50	0.60	4.37	0.65	4.30	0.85	4.22	0.71	3.78	0.85	3.76	0.94	3.76	1.02
	*P value*	0.089		0.055		0.561		0.386		0.328		0.432		0.122		0.419		0.459		0.648	
Length of stay	< 9 days	4.4	0.54	4.2	0.7	4.52	0.53	4.45	0.59	4.39	0.62	4.33	0.7	4.1	0.84	3.99	0.84	3.93	0.86	3.88	1.01
	9 to 13 days	4.52	0.59	4.43	0.81	4.53	0.62	4.66	0.59	4.49	0.58	4.6	0.5	4.16	0.99	4.24	0.69	3.86	0.95	3.92	1.
	> 13 days	4.4	0.62	4.16	0.76	4.43	0.73	4.46	0.64	4.15	0.59	4.4	0.8	4.14	0.72	3.79	0.84	3.87	0.83	3.64	0.96
	*P value*	0.621		0.103		0.513		0.567		0.05		0.129		0.386		0.25		0.82		0.665	
Age group	< 50 years	4.17	0.71	4.02	0.85	4.36	0.69	4.15	0.66	4.04	0.61	4.32	0.6	4.03	0.66	3.85	0.66	3.63	0.94	3.65	1.02
	50–65 years	4.47	0.55	4.28	0.76	4.55	0.61	4.57	0.53	4.39	0.66	4.27	0.93	3.97	0.9	3.93	0.88	3.97	0.87	3.86	1.
	> 65 years	4.51	0.48	4.3	0.67	4.52	0.57	4.61	0.57	4.44	0.55	4.51	0.59	4.25	0.85	4.05	0.86	3.99	0.81	3.87	0.98
	*P value*	***0.047***		***0.04***		0.185		***0.048***		***0.046***		0.195		0.241		0.329		***0.009***		0.175	

**µ**-> Mean of distribution; **σ**-> Standard deviation; HCAs (Health Care Assistants).

For the variable ‘respondents’, patients gave higher mean scores for the domains: department’s image (µ = 4.49, p = 0.001) and medical care (µ = 4.51; p = 0.014) than patient representatives. In the variable ‘education level’, respondents with a medium/higher level of education (secondary or university education) gave lower mean scores in the domains: HCAs (µ = 4.17; p = 0.013), reception (µ = 3.89; p = 0.002), food (µ = 3.74; p = 0.004), diagnostic tests (µ = 3.72; p = 0.04) and transport (µ = 3.62; p = 0.025). In the variable ‘age group’, respondents < 50 years old, assessed the mean score in the domains: department’s image (µ = 4.17; p = 0.047), facilities (µ = 2.4; p = 0.04), nurse care (µ = 4.15; p = 0.048), HCAs (µ = 4.04; p = 0.046) and diagnostic tests (µ = 3.63; p = 0.009).

Questionnaire Raw data showing characteristics of patients, respondents and their responses to the questionnaire.

## Discussion

The typical participant was male, married and retired with a low educational level in this convenience sample. Respondents reported good results with the care provided from the professionals, with scores above four points, but the amenities were rated below this score. The present findings seem to be consistent with another study ^7^, which found the following factors to be scored, in descending order: medical performance, nursing staff, amenities and accessibility. In another study in Israel ^8^, the attitudes of nurses and medical care were the most important determinants of patient satisfaction with the care received. In a study performed in Kuwait ^9^, medical care was the most favorably rated domain, followed by admission process and housekeeping, while nursing care was the least favorably rated domain. It can therefore be assumed that the assessment of patient’s satisfaction is based not only on the care received.

The evaluation instrument used here was a questionnaire developed for this purpose, following a search of the literature on the satisfaction of patients or families to determine the applicability of the questions used. Some studies ^4– 10^ have chosen variables related to information provided from the patient or their family, the existing support structures, the services available at the hospital and concerns about the ability to meet the patient’s needs during hospitalization.

To avoid bias in the questionnaire ^4^, we used two methods: peer review for content validation and prior testing by a group of inpatients. To test the reliability of the questionnaire we used Cronbach’s alpha, and the results showed values indicating internal consistency in all areas. If alpha is too high this may suggest that some items are redundant ^6^ and test the same question but in a different way. A maximum alpha value of 0.90 is recommended ^6^.

The response rate of 78% for a sample of convenience and a self-administered questionnaire is considered good. This finding is in agreement with Stizia’s ^11^ findings, which showed a response rate of 65% for self-administered questionnaires and in studies with a convenience sample of 25%.

In this study, we found that gender, marital status, occupation (retired or not) and length of stay did not affect the scores in the domains of the questionnaire. A previous study has shown that older subjects tend to have higher scores ^12^, and this was confirmed in all the areas of our questionnaire.

Data from this study showed that the higher the educational level, the lower the scores for the responses in the domains of amenities, with the exception of medical care. A possible explanation for these differences might be better understanding and knowledge of the procedures to be undergone. Our study is in agreement with another ^4^ in which had an inverse relation to educational status, with high educational levels associated with low scores.

We found that with the variable ‘respondent’, patients provided high scores in all domains, with statistical significance in the medical care. Unlike the inpatients, the representatives tended to be more demanding or critical and reported lower scores in some domains. It is likely that representatives complain about inadequate information and practical advice, especially on how to deal with potential decompensated disease ^13^.

This study provides valuable information on the effect of all variables in the various fields that constitute our satisfaction tool in hospitalized patients. Therefore, we offer a picture of the determinants of satisfaction in several areas, which have never been studied together. The study may contribute to the understanding of the factors that influence inpatient satisfaction in hospital wards and could be used as a resource for evaluating the quality of care.

The limitations of this study include that it is a cross-sectional study and inevitably a convenience sample, composed of the patients discharged during one month. However, this convenience sampling is useful when attempting to study the satisfaction of a niche segment such as inpatients in internal medicine. Moreover, the range of possible explanatory variables included in this study, although large, was not as comprehensive as we would have liked. Several other variables, such as previous health status, could also have been assessed. However, when previous mental and physical health is poor, this is associated with lower satisfaction with hospitalization ^14^. These patients have more hospitalizations, more aggressive treatment, and are more likely to suffer from medical complications ^15^.

## Conclusions

This study allowed us to conclude that respondent satisfaction was above average in all areas of the questionnaire. Although the mean score was high for all domains, amenities had the lowest level of satisfaction, pointing to the need to reassess food, transport and diagnostic tests. Gender, marital status and length of stay at the hospital were not factors that influenced the level of satisfaction of respondents.

Despite the limitations of the study, it was possible to identify the level of satisfaction of patients hospitalized in the internal medicine department, and the influential variables. These findings will allow the hospital management to implement changes in practice and propose actions to improve quality of care, and provide visibility to the teamwork of professionals involved. We conclude that, as in previous studies, there is evidence that the educational level, age and amenities affect the levels of satisfaction. Finally, we must consider that patient representatives are more critical than patients in the evaluation of satisfaction. Therefore, researchers conducting a survey of hospitalized patients’ satisfaction in internal medicine departments should be aware of the effect of variables on the responses to the questionnaire and make the necessary adjustments to provide valid results.

## Figures and Tables

**Satisfaction Questionnaire (sheet 1):**
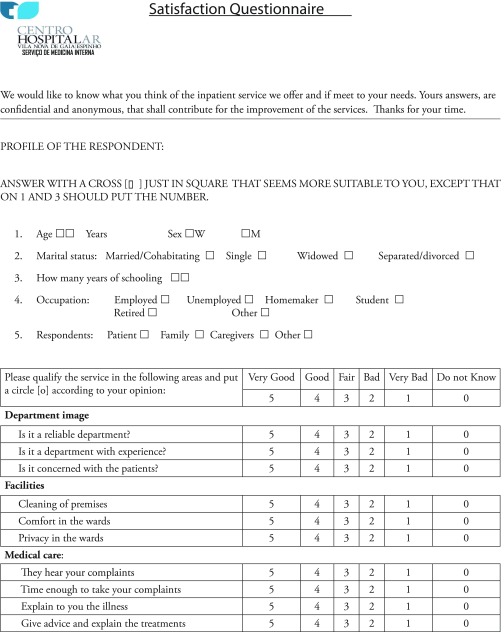
Questionnaire handed out to study participants (English translation).

**Satisfaction Questionnaire (sheet 2):**
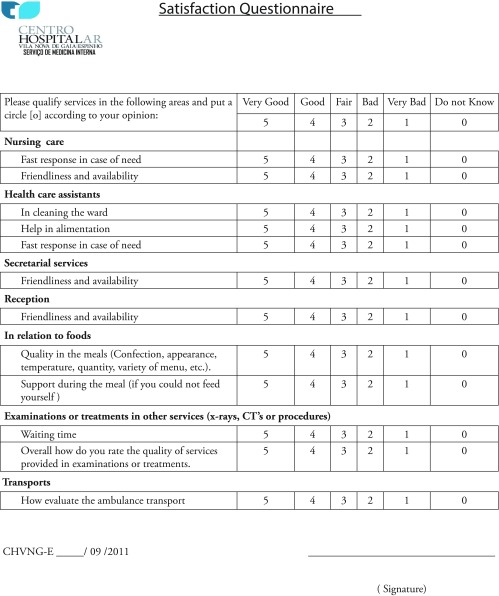
Questionnaire handed out to study participants (English translation).

**Satisfaction Questionnaire (sheet 1):**
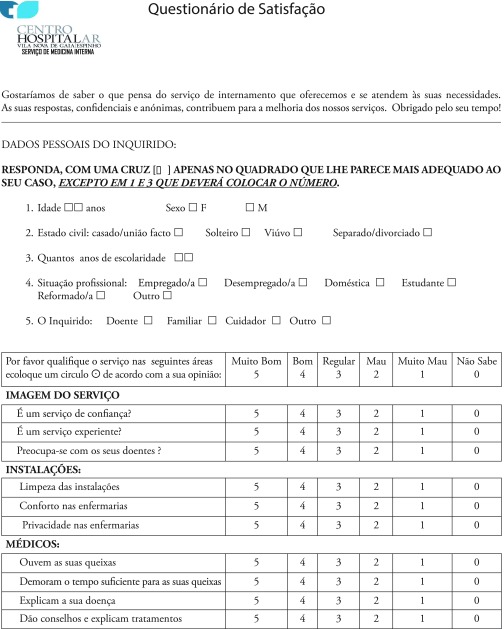
Questionnaire handed out to study participants (Portuguese translation).

**Satisfaction Questionnaire (sheet 2):**
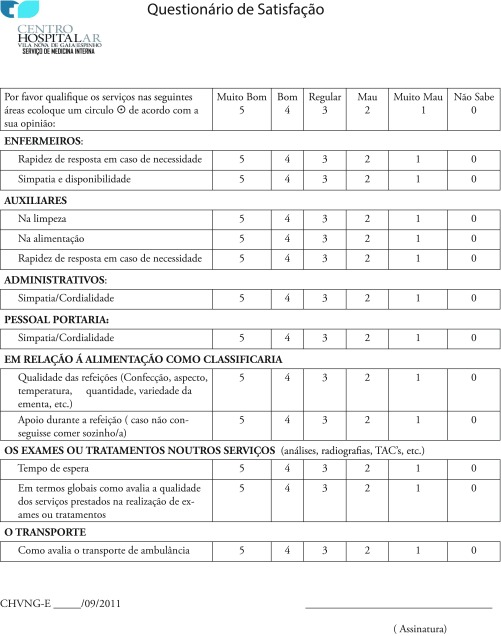
Questionnaire handed out to study participants (Portuguese translation).
