# Supplementary material for: Inpatients’ opinions on a hospital in Portugal
Source: F1000Res. 2013 Feb 13;2:49. [Version 1] doi: 10.12688/f1000research.2-49.v1 (PMC3901457; doi:10.12688/f1000research.2-49.v1)
Supplement: Peer review history

# Peer review: Referee response for version 1

**Published:** 2013-05-31

**Authors:** Saravana Kumar

**Affiliations:** 1 Division of Health Sciences, University of South Australia, Adelaide, Australia

This is an interesting and well conducted research study which sheds light on the patients’ perspectives of aspects of health care in an internal medicine ward. It is a well presented manuscript which is easy to read with logically presented arguments. I have two main comments.

1. It would have been worthwhile to expand on the process which led to the development of the survey instrument. While the authors provide a brief overview of the process, such as the conduct of the focus groups etc, it is imperative to provide detailed information about this as the development and the psychometric properties of the instrument can play an important role in the believability of the research findings. 

2. I am not sure about some of the items in the survey instrument. For example, what does “is it a reliable department” actually mean? Reliable in term so what? Similarly, what does “is it a department with experience” actually relate to. Is it a question about health practitioners who work there or about how long the department has been in place and hence it reputation? These questions may give rise to ambiguity in the interpretation and as such influence the responses.

I have read this submission. I believe that I have an appropriate level of expertise to confirm that it is of an acceptable scientific standard.

# Peer review: Referee response for version 1

**Published:** 2013-02-22

**Authors:** Erin Aiello Bowles

**Affiliations:** 1 Research Department, Group Health Research Institute, Seattle, WA, USA

I only have one major comment, which is that the authors don't provide any information on non-responders. It would be interesting to know more about the characteristics of those people, and whether they differ from responders because this could affect the generalizability of the results.

I have read this submission. I believe that I have an appropriate level of expertise to confirm that it is of an acceptable scientific standard.

# Response

**Published:** 2013-03-14

**Authors:** Antonio Agripino Costa Oliveira

**Affiliations:** Centro Hospitalar Gaia-Espinho, Portugal

We want to express our appreciation to Erin Aiello Bowles for taking the time and effort necessary to revise our manuscript. We have carefully considered your comment which states that we didn't provide any information on non-responders. We agree that sampling bias is the major problem in patient satisfaction studies; but in our study the response rate was high and the self-administered questionnaire was filled out by patients or their representatives at time of discharge. Indeed we have some information on the variables; sex, age and length of stay, in patient non-responders, but we don´t have any information for the representative non-responders.
